# TrkB expression and dependence divides gustatory neurons into three subpopulations

**DOI:** 10.1186/s13064-019-0127-z

**Published:** 2019-01-28

**Authors:** Jennifer Rios-Pilier, Robin F. Krimm

**Affiliations:** Department of Anatomical Sciences and Neurobiology, University of Louisville School of Medicine, 511 S. Floyd St., MDR Building Room 111, Louisville, KY 40202 USA

**Keywords:** BDNF, TrkB, Neurotrophin, Taste, Geniculate ganglion, Taste buds, Innervation, Neuron development, Gustatory

## Abstract

**Background:**

During development, gustatory (taste) neurons likely undergo numerous changes in morphology and expression prior to differentiation into maturity, but little is known this process or the factors that regulate it. Neuron differentiation is likely regulated by a combination of transcription and growth factors. Embryonically, most geniculate neuron development is regulated by the growth factor brain derived neurotrophic factor (BDNF). Postnatally, however, BDNF expression becomes restricted to subpopulations of taste receptor cells with specific functions. We hypothesized that during development, the receptor for BDNF, tropomyosin kinase B receptor (TrkB), may also become developmentally restricted to a subset of taste neurons and could be one factor that is differentially expressed across taste neuron subsets.

**Methods:**

We used transgenic mouse models to label both geniculate neurons innervating the oral cavity (Phox2b+), which are primarily taste, from those projecting to the outer ear (auricular neurons) to label TrkB expressing neurons (*TrkB*^GFP^). We also compared neuron number, taste bud number, and taste receptor cell types in wild-type animals and conditional TrkB knockouts.

**Results:**

Between E15.5-E17.5, TrkB receptor expression becomes restricted to half of the Phox2b + neurons. This TrkB downregulation was specific to oral cavity projecting neurons, since TrkB expression remained constant throughout development in the auricular geniculate neurons (Phox2b-). Conditional TrkB removal from oral sensory neurons (Phox2b+) reduced this population to 92% of control levels, indicating that only 8% of these neurons do not depend on TrkB for survival during development. The remaining neurons failed to innervate any remaining taste buds, 14% of which remained despite the complete loss of innervation. Finally, some types of taste receptor cells (Car4+) were more dependent on innervation than others (PLCβ2+).

**Conclusions:**

Together, these findings indicate that TrkB expression and dependence divides gustatory neurons into three subpopulations: 1) neurons that always express TrkB and are TrkB-dependent during development (50%), 2) neurons dependent on TrkB during development but that downregulate TrkB expression between E15.5 and E17.5 (41%), and 3) neurons that never express or depend on TrkB (9%). These TrkB-independent neurons are likely non-gustatory, as they do not innervate taste buds.

## Background

The geniculate ganglion primarily contains two neuron subpopulations: 1) neurons that carry mostly taste, but some somatosensory information from the anterior two-thirds of the tongue and the palate to the brain (oral sensory), and 2) those that innervate the outer ear through the auricular nerve (auricular neurons). Development of most of these neurons is regulated by the neurotrophin brain-derived neurotrophic factor (BDNF). BDNF binds with high affinity to the tropomyosin kinase B receptor (TrkB) [1, 2] and critically regulates the survival of taste neurons [3, 4]. Early in development, BDNF expression in taste buds acts as a cue for TrkB+ fibers to innervate taste organs [5–12]. Both this target innervation process and neuron survival occur during a critical developmental period [8, 10]. After this critical period, BDNF is downregulated in the taste bud, but continues to be expressed in a subpopulation of taste receptor cells [13, 14]. This expression change suggests that BDNF may play a different role/s in later developmental stages and adulthood.

Most geniculate ganglion neurons express the BDNF receptor TrkB [15–17] early in development and depend on TrkB signaling for their survival [15, 18, 19]. However, in TrkB knockout animals, many innervated taste buds remain at birth despite profound geniculate neuron loss by E13.5 [15, 18]. One possible explanation is that gustatory neurons lacking the TrkB receptor could migrate into the geniculate ganglion after E13.5. Consistently, in chick embryos, geniculate neurons continue to delaminate from the epibranchial placodes and migrate into the geniculate ganglion throughout embryonic development [20]. How many gustatory neurons remain after E13.5 in TrkB knockouts is unclear, particularly because earlier studies lacked markers for taste neurons and did not quantify geniculate neurons after E13.5 [15, 18]. Therefore, a subset of TrkB-independent taste neurons of unknown size likely exists.

By adulthood, only a subset of taste neurons appears to be regulated by BDNF, as adult BDNF removal only reduces taste bud innervation by 40% [21, 22]. Therefore, in adulthood, many gustatory neurons likely lack the TrkB receptor. Yet, it remains unknown if, when, or how, during development or adulthood, TrkB expression decreases in the neurons that innervate the oral cavity. Here, our primary goals were to determine 1) how many neurons express TrkB in adulthood, and 2) when and how TrkB expression decreases during development.

Previous studies examining TrkB expression in adulthood lacked markers for distinguishing the roughly 50% of geniculate ganglion neurons that are oral sensory from the auricular neurons innervating the outer ear. The transcription factor Phox2b was recently established as a marker that distinguishes geniculate ganglion neurons innervating the oral cavity (Phox2b+) from auricular neurons (Phox2b-) [23, 24]. Using Phox2b as a marker for geniculate oral sensory neurons, we determined that developmental reduction of TrkB-expression and differential dependence on TrkB for survival divide oral sensory neurons of the geniculate into three subsets. These subpopulations were: 1) neurons that depend on TrkB expression during development and continue to express TrkB receptors in adulthood (50%), 2) neurons that depend on TrkB during development but downregulate it between E15.5 and birth (41%), and 3) neurons that never express or depend on TrkB and do not innervate taste buds (9%). Therefore, adult roles of TrkB-signaling are likely restricted to a subset of oral cavity projecting neurons.

## Methods

### Animals

All mice were housed in a central facility and maintained under controlled conditions of normal humidity and temperature, with standard alternating 12-h periods of light/dark and free access to water and food. *Animals were cared for and used in accordance with guidelines of the U.S. Public Health Service Policy on Humane Care and Use of Laboratory Animals and NIH Guide for the Care and Use of Laboratory Animals.*

To visualize TrkB expression in Phox2b-expressing neurons, Phox2b-Cre (Tg [Phox2b-cre]NP91Gsat/Mmucd, Stock No. 034613-UCD [23]) with tdTomato (Ai14, Jax Stock No. 007914) mice were bred with *TrkB*^GFP/+^ (B6.129S6[Cg]-*Ntrk2*^*tm2.1Ddg*^/J, Jax Stock No. 023046) mice [25, 26]. In Phox2b-Cre mice, gene recombination occurs in any neuron that has ever expressed Phox2b. In the geniculate ganglion, this is specific to oral sensory neurons and excludes the somatosensory neurons that innervate the outer ear via the auricular nerve [23]. These oral sensory neurons are mostly gustatory, but also include somatosensory fibers [24, 27–29]. However, most of the somatosensory neurons innervating the tongue arise from the trigeminal ganglion. In *TrkB*^GFP/+^ mice, GFP had been shown to be expressed in the same geniculate neurons that labeled with an anti-TrkB antibody ([27], supplementary Fig. 3), making it an appropriate marker for TrkB-expressing neurons.

Embryo heads from mice aged E13.5 (*n* = 2), E15.5 (*n* = 3), E17.5 (*n* = 4), and P0 (n = 3) were collected for immunohistochemistry. The day that a vaginal plug was observed was designated E0.5. Geniculate ganglia from adult mice (P60) were also collected for whole mount and serial sections. To conditionally remove the TrkB receptor from oral cavity neurons, we bred Phox2b-Cre::tdTomato::*TrkB*^GFP/+^ mice with a mouse line in which the coding region of the *TrkB* gene was surrounded by loxP sites (*Ntrk2*^*tm1Ddg*^/J mouse, Jax Stock No. 022363) [30]. These conditional TrkB knockout mice do not live to adulthood and instead die at different postnatal ages [15, 31], so we collected geniculate ganglia and tongues from Phox2b-Cre::tdTomato::*TrkB*^GFP/loxP^ (*n* = 8) and Phox2b-Cre::tdTomato::*TrkB*^GFP/+^ (*n* = 5) P20. These geniculate ganglia were processed to determine how many oral sensory neurons depended on TrkB signaling during development.

### Immunohistochemistry

Embryos aged E13.5 were fixed by immersion in 4% paraformaldehyde (PFA). Embryos aged E15.5 and older, young (P20) mice, and adult (P60) mice were all sacrificed with an overdose (1 ml, i.p.) of 2.5% tribromoethanol (Avertin) and then were trans-cardially perfused with 4% PFA. Geniculate ganglia were dissected under a microscope and then embryo and newborn heads or geniculate ganglia from adult mice were post-fixed in 4% PFA overnight at 4 °C. Tissue was then transferred to 30% sucrose/phosphate-buffered saline (PBS) for cryoprotection at 4 °C overnight. The following day, tissue was frozen on dry ice in OCT (Sakura Tek) embedding medium and stored at − 80 °C until processed for immunohistochemistry.

To visualize oral cavity neurons in adult mice, entire geniculate ganglia were rinsed in 0.1 M PB four times and blocked overnight with 3% donkey serum in 0.1 M PBS containing 0.5% Triton X-100 (PBST). Ganglia were then incubated with the following primary antibodies for 5 d at 4 °C: goat anti-GFP (1:400; Novus, AB Registry ID: AB_10128178, Cat No. NB100–1700, Littleton, CO) and rabbit polyclonal anti-dsRed (1:500, Clontech Laboratories, Inc., Cat No. 632496, Mountain View, CA) and for some ganglia anti-calbindin (1:1000; Neuromics, Edina, MD). After incubation in primary antibodies and four rinses with 0.1 M phosphate buffer (PB), tissues were incubated for 2 d in the following secondary antibodies: anti-goat Alexa Fluor 488 (1:500; Jackson ImmunoResearch Laboratories, West Grove, PA) and anti-rabbit Alexa Fluor 555 (1:500; Jackson ImmunoResearch Laboratories) and for some ganglia anti-chicken Alexa 647 (1:500; Jackson ImmunoResearch Laboratories). The tissue was then washed four times in 0.1 M PB, mounted onto slides, and cover-slipped using aqueous mounting medium (Fluoromount-G, SouthernBiotech, Birmingham, AL).

Alternatively, serial transverse sections (20 μm) were cut using a cryostat. Sections were left to air-dry on a slide warmer overnight. The next day, sections were post-fixed in 4% PFA for 15 min at 4 °C. After four rinses with PBST, sections were blocked for 1 h at room temperature with 5% normal goat serum in PBST. Then, the tissue was incubated overnight at 4 °C with the following primary antibodies: chicken anti-GFP (1:1000; Thermo Fisher, Cat No. A10262, Carlsbad, CA) and rabbit anti-P2X3 (1:500; Millipore, AB Registry ID: AB_11212062, Cat No. AB58950, Billerica, MA). After incubation in primary antibodies and four rinses in PBST, sections were incubated for 1 h at room temperature in the following secondary antibodies: goat anti-chicken Alexa Fluor 488 (1:500; Jackson ImmunoResearch Laboratories) and goat anti-rabbit Alexa Fluor 647 (1:500; Jackson ImmunoResearch Laboratories). The tissue was then washed four times in 0.1 M PBS, mounted onto slides, and cover-slipped using aqueous mounting medium (Fluoromount-G).

To visualize fungiform papillae and innervated taste buds, anterior tongue-halves from Phox2b-Cre::tdTomato::*TrkB*^GFP/loxP^ and Phox2b-Cre::tdTomato::*TrkB*^GFP/+^ mice were collected at P20 using the same procedures as described for geniculate ganglia. To examine taste bud innervation and Car4-positive taste receptor cells, thick sagittal sections (70 μm) of one half tongue from each animal were cut using a cryostat and rinsed in 0.1 M PB four times for 15 min. After blocking with 3% normal donkey serum in 0.1 M PB containing 0.5% Triton X-100 overnight at 4 °C, tissues were incubated for 5 d in the following primary antibodies: rat anti-cytokeratin-8 (K8, 1:50, Cat No. Troma-1-s, Developmental Studies Hybridoma Bank, Iowa City, IA), rabbit polyclonal anti-dsRed (1:500, Clontech Laboratories, Inc., Cat No. 632496), and either goat anti-Car4 (1:500, Cat No. AF2414, R&D Systems, Minneapolis, MN). Floating sections were rinsed in 0.1 M PB four times for 15 min and incubated for 2 d in the following secondary antibodies (Jackson ImmunoResearch Laboratories): donkey anti-rat Alexa Fluor 488, donkey anti-rabbit Cy3, and donkey anti-goat Alexa 647. To examine taste bud innervation and PLCβ2-positive taste receptor cells, the other half of the tongue for each animal was incubated for 5 d in rabbit anti-PLCβ2 (1:500, Cat No. SC-206, Santa Cruz Biotechnology, Dallas, TX). Floating sections were rinsed in 0.1 M PB four times for 15 min and incubated for 2 d in donkey anti-rabbit Alexa Fluor 647. Then, sections were rinsed in 0.1 M PB four times for 15 min and blocked for 2 d with donkey anti-rabbit (1:100, Cat No. 007–003-007, Jackson ImmunoResearch, Laboratories). After four rinses in 0.1 M PB for 15 min, sections were incubated for 5 d in the following primary antibodies: rat anti-K8 (1:50, Cat No. Troma-1-s, Developmental Studies Hybridoma Bank, Iowa City, IA), and rabbit polyclonal anti-dsRed (1:500, Clontech Laboratories, Inc., Cat No. 632496). Floating sections were rinsed in 0.1 M PB four times for 15 min and incubated for 2 d in the following secondary antibodies (Jackson ImmunoResearch Laboratories): donkey anti-rat Alexa 488, donkey anti-goat Alexa Fluor 488, and donkey anti-rabbit Cy3. To quantify fungiform papillae, all tissues were then rinsed in 0.1 M PB and stained with DAPI (2 μL in 50 mL of double distilled H20, Life Technologies, Foster City, CA) for 45 min. After four rinses in 0.1 M PB, tissues were cover-slipped using aqueous mounting medium (Fluoromount-G).

### Neuron quantification of whole mounts at P20 and in adulthood

Images of whole ganglia were captured using a 40× oil immersion lens (FV1200, Olympus) with a step size of 0.47 μm. Confocal images were obtained by stitching multiple fields to create a high-resolution image of the entire ganglia in a single Z-stack. Each labeled channel was collected individually using single-wavelength excitation. Images of each optical section through the whole ganglia were then analyzed using Neurolucida 360 (MBF Bioscience, Williston, VT). Brightness and contrast were adjusted for background standardization in all images. Each labeled neuron was followed through the optical sections so that each neuron was only counted once. The absolute number of single- (anti-dsRed) and double-labeled (anti-dsRed and anti-GFP) neurons were counted by examining each cell through the Z-stack. Neuron size was measured by measuring the area of the neurons from the optical section where the neuron appears to be its largest size; diameters were then calculated from areas.

### Neuron quantification of sections during development

Serial sections of embedded embryo and newborn heads and adult geniculate ganglia were captured using a 40× oil immersion lens (FV1200, Olympus). Confocal images were collected using single-wavelength excitation taken with a step size of 1.0 μm. Alternating images from serial sections containing ganglia were then analyzed using Neurolucida 360 (MBF Bioscience). The number of single- (anti-dsRed) and double-labeled (anti-dsRed and anti-GFP) neurons were counted in alternating sections by following each cell through the Z-stack so that each neuron was only counted once. The percentage of oral cavity neurons (tdTomato+) expressing TrkB (GFP+) was calculated for each section and averaged for each animal per age group and then compared to tdTomato single-positive neurons. To quantify TrkB expression in non-taste neurons, the percentage of neurons that expressed both TrkB (GFP) and P2X3 but not Phox2b was examined across ages.

### Quantification of fungiform papillae and taste bud number after conditional removal of TrkB from oral sensory neurons

Images of taste buds were captured from thick sections of tongue-halves using a 60× oil immersion lens with a step size of 1.0 μm. Images were collected using single-wavelength excitation and projected along the Z-axis. To determine how TrkB removal affected taste bud number, we counted taste buds in fungiform papillae using DAPI staining with a fluorescence microscope and a 20× objective lens. We examined all sections for one-half of a mouse tongue. Anti-K8 labeling indicated the presence of a taste bud in each fungiform papilla. We examined all the taste buds labeled with anti-K8 on each half tongue and quantified the number of taste buds containing Car4+ or PLCβ2+ labeled cells. The percentage of taste buds containing Car4+ and PLCβ2+ taste receptor cells was calculated by dividing the number of taste buds containing labeled cells by the total number of taste buds for each one-half of tongue (anti-K8, *n* = 3/genotype).

### Data analysis

A student’s t-test was used to compare the number of double-labeled vs. single-labeled neurons in Phox2b-Cre::tdTomato::*TrkB*^GFP/lox^ mice in adulthood (*n* = 3). A Chi-squared (X^2^) test was used to analyze the percentage of Phox2b neurons that express TrkB in geniculate ganglion sections in E15.5 (*n* = 3), compared to E17.5 (*n* = 4), P0 (n = 3) and adult (n = 3). Because this is 3 statistical tests, type one error was controlled using a Bonferroni correction, and so a *p*-value of *p* < 0.0167 was established before analysis. A Chi-squared (χ^2^) test was also used to analyze TrkB expression in Phox2b-negative neurons across different ages (E13.5 (*n* = 2), P0 (*n* = 3), and adult (n = 3)) and Bonferroni correction was used for a *p*-value of *p* < 0.025. A students t-test was used to compare the number of remaining neurons at P20 in conditional TrkB knockouts (Phox2b-Cre::tdTomato::*TrkB*^GFP/lox^, *n* = 8) compared with controls (Phox2b-Cre::tdTomato::*TrkB*^GFP/+^, controls, *n* = 5). A student’s t-test was used to compare the number of taste buds between controls (Phox2b-Cre::tdTomato::*TrkB*^GFP/+^, n = 5) and conditional knockouts (Phox2b-Cre::tdTomato::*TrkB*^GFP/lox^ n = 5). Chi-squared (χ^2^) test was used to compare the percentage of taste buds with PLCβ2+ and Car4+ cells between (Phox2b-Cre::tdTomato::*TrkB*^GFP/lox^, conditional knockouts, *n* = 3) and controls (Phox2b-Cre::tdTomato::*TrkB*^GFP/+^, controls, n = 3). Except where otherwise specified, a significance for all tests was established as *p* < 0.05 before analysis; however, actual *p*-values are reported.

## Results

### Only half of the oral sensory neurons in the geniculate ganglion express TrkB in adulthood

Early in development, approximately 90% of geniculate neurons express TrkB and depend on TrkB for their survival [15, 18]. We hypothesized that following this critical period for neuron survival, only a subset of the neurons projecting to the anterior two-thirds of the tongue would continue to express TrkB in adulthood. This would be consistent with the finding that not all adult gustatory nerve fibers appear to express TrkB in adulthood [22].

**Fig. 1:**
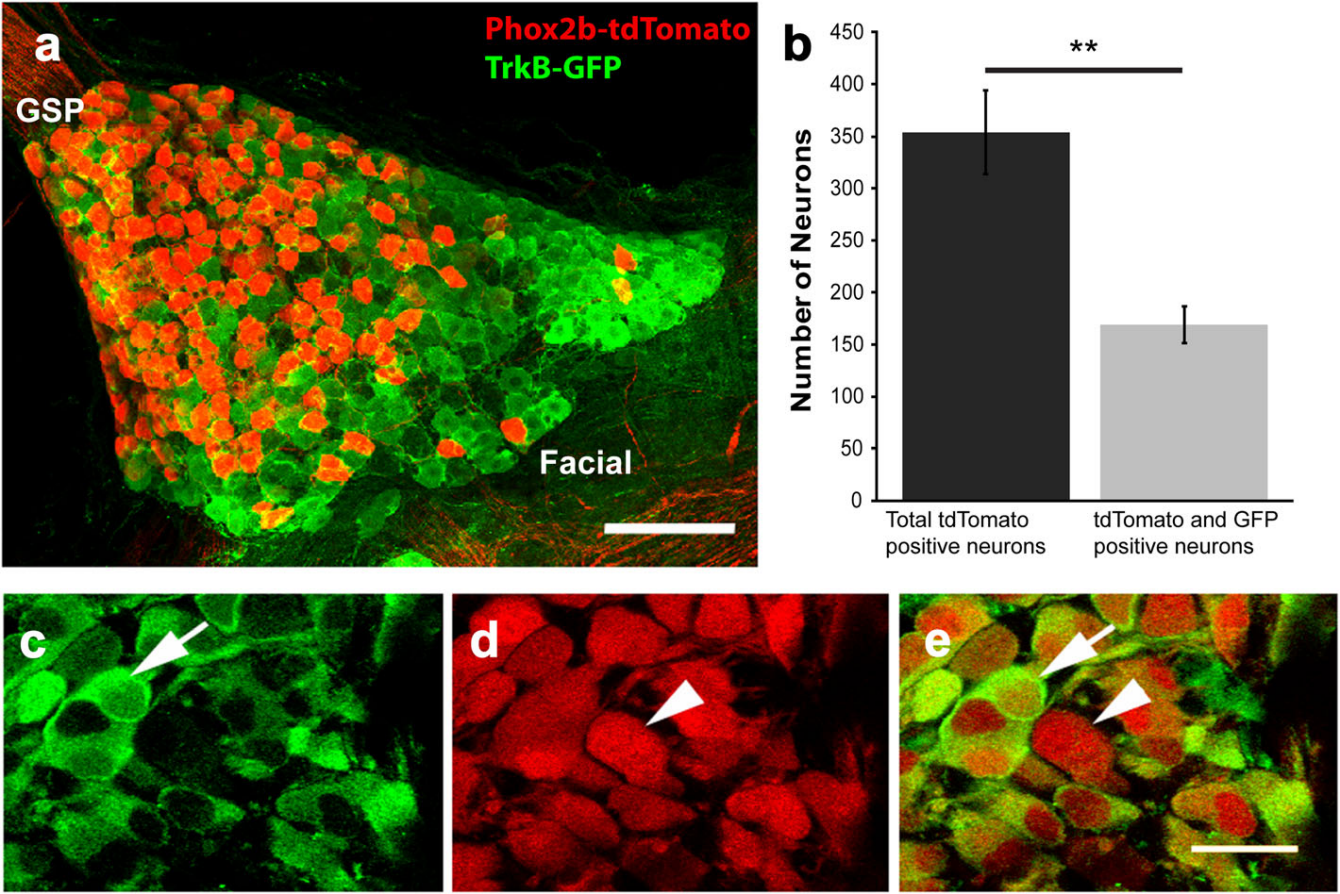
Half of adult taste neurons express the TrkB receptor. **a**) Representative image of the entire z-stack of a whole mount adult geniculate ganglion from a Phox2b-Cre::tdTomato::*TrkB*^GFP/+^ mouse labeled for both tdTomato (oral cavity-projecting, red) and GFP (TrkB, green). **b**) Quantification of the total number of neurons (*n* = 3) projecting to the oral cavity (red only) and oral cavity-projecting neurons that express *TrkB*^GFP^ (double-labeled). **c**-**e**) Magnified images of a single optical section illustrate the criteria used to identify and count single-labeled (arrowhead) and double-labeled (arrow) oral cavity-projecting neurons. Scale bar = 50 μm (**a**) and 10 μm (**c**-**e**). ***p < 0.001

Recently, Phox2b expression has been used to distinguish oral sensory neurons (Phox2b+) from those innervating the outer ear (auricular neurons; Phox2b-) [23, 24]. Trigeminal neurons innervating the tongue are also Phox2b-. Thus, in the geniculate ganglion, Phox2b expression is fairly specific to taste neurons, plus a few general oral sensory neurons. To visualize TrkB expression in oral sensory neurons, we crossed Phox2b-Cre::tdTomato mice with *TrkB*^GFP/+^ mice to visualize TrkB receptor expression (GFP+: green) in oral sensory neurons (dsRed+, red; Fig. 1a). We quantified neurons from Z-stacks of adult (P60) whole mount geniculate ganglia. In adulthood, not all geniculate ganglion neurons expressed the TrkB receptor (Fig. 1a, b). Specifically, while GFP labeling was distributed throughout the ganglion, tdTomato+ neurons were located more medially and closer to the greater superficial petrosal nerve (Fig. 1a). This suggests that oral cavity-projecting neurons are primarily localized to one part of the ganglion.

We then quantified the numbers of single-labeled (TrkB-, tdTomato only) and double-labeled (TrkB+, red and green) oral cavity-projecting neurons in whole mount P60 geniculate ganglia (Fig. 1b). An average of 354 ± 33 neurons were Phox2b+. Of those, 185 ± 21 neurons co-labeled with GFP, while 169 ± 0 neurons expressed tdTomato alone (**p < 0.01; Fig. 1b). Thus, roughly 52% of oral cavity-projecting neurons express TrkB in adulthood. Although different in terms of TrkB expression these two populations were not different in terms of diameter (Phox2b-only (17.8 ± 0.2) vs Phox2b/TrkB (17.7 ± 0.2), *p* = 0.21).

### TrkB expression declines between E15.5 and E17.5

**Fig. 2:**
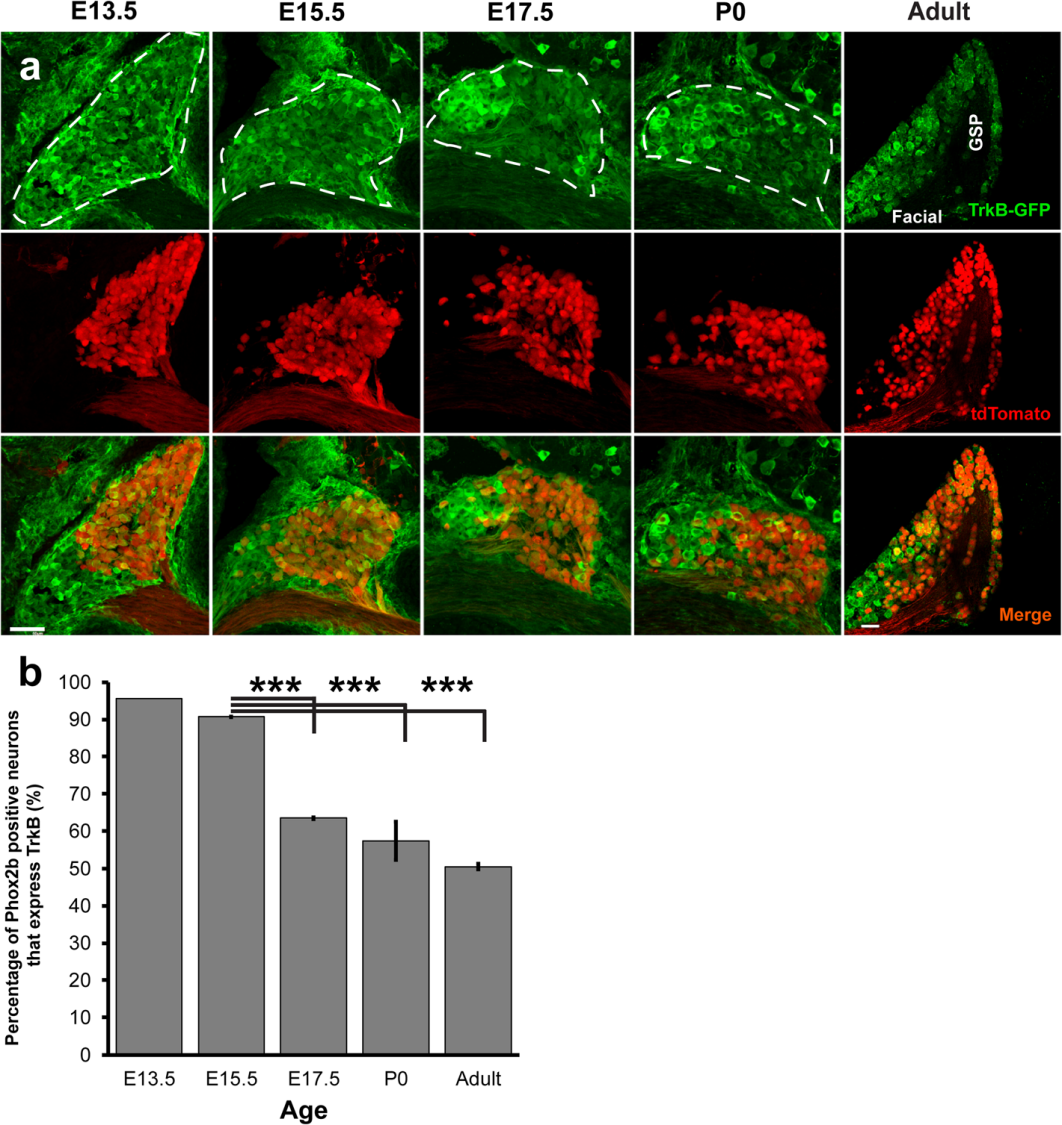
TrkB expression decreases before birth in oral cavity-projecting neurons and remains reduced through adulthood. **a**) Representative Z-stack images of TrkB-GFP (green, top) and Phox2b-tdTomato (red, middle) from geniculate ganglion sections across ages E13.5 (*n* = 2), E15.5 (*n* = 3), E17.5 (*n* = 4), P0 (*n* = 3), and P60 (*n* = 3). Dashed lines illustrate the boundaries of the geniculate ganglion. **b**) Mean percentage ± SEM of Phox2b-positive neurons that express TrkB receptor during development and adulthood. ***p < 0.001, Scale bar = 50 μm

Although approximately half of oral cavity-projecting neurons express TrkB in adulthood (Fig. 1), approximately 90% of geniculate neurons express TrkB between E11.5 and E13.5 [15–17, 32]. We next sought to determine at what period during development TrkB expression decreases. Using Phox2b-tdTomato to identify oral cavity-projecting neurons, we quantified the number of tdTomato+ neurons expressing TrkB-GFP at ages E13.5, E15.5, E17.5, at birth (P0), and adulthood (P60) from serial transverse sections using immunohistochemistry. Consistent with previous findings [15, 17], we observed *TrkB*^GFP^ labeling throughout most of the geniculate ganglion at E13.5. *TrkB*^GFP^ labeling was also observed outside of the geniculate ganglion (Top panel, Fig. 2a-e). Across all ages examined, Phox2b-tdTomato labeling (red) was more prevalent in the medial portion of the geniculate ganglion (Middle panel, Fig. 2a-e). At E13.5 and E15.5, *TrkB*^GFP^ labeling appeared uniformly distributed throughout the geniculate ganglion. After E17.5, TrkB labeling appeared to be more strongly expressed tdTomato- (non-taste) neurons, and this pattern persisted through adulthood (bottom panel, Fig. 2a-e).

Understanding the temporal dynamics of TrkB expression might provide insight into the mechanism underlying decreased TrkB expression in adulthood. To determine when during development TrkB expression begins to decrease in oral sensory neurons, the percentage of Phox2b + neurons co-labeled with GFP was quantified E13.5-P60 (Fig. 2f). At E13.5, most (95%) tdTomato+ neurons expressed TrkB. The remaining 5% of tdTomato+ neurons likely consisted of the small subpopulation of TrkB-independent geniculate ganglion taste neurons [15]. TrkB expression in oral cavity-projecting neurons did not differ between ages E13.5 and E15.5 (91%; *p* = 0.151). However, by E17.7, TrkB expression was significantly reduced to only 63% (***p < 0.001) and remained reduced at birth (57%; ***p < 0.001). By adulthood, only 50% of tdTomato+ neurons expressed GFP. These data replicate our findings regarding TrkB expression in whole mount ganglia (Fig. 1). Thus, TrkB expression is significantly reduced to approximately 50% of oral cavity-projecting neurons between E15.5 and E17.5 and remains reduced into adulthood.

### Decreased TrkB expression is specific to Phox2b + oral sensory neurons

**Fig. 3:**
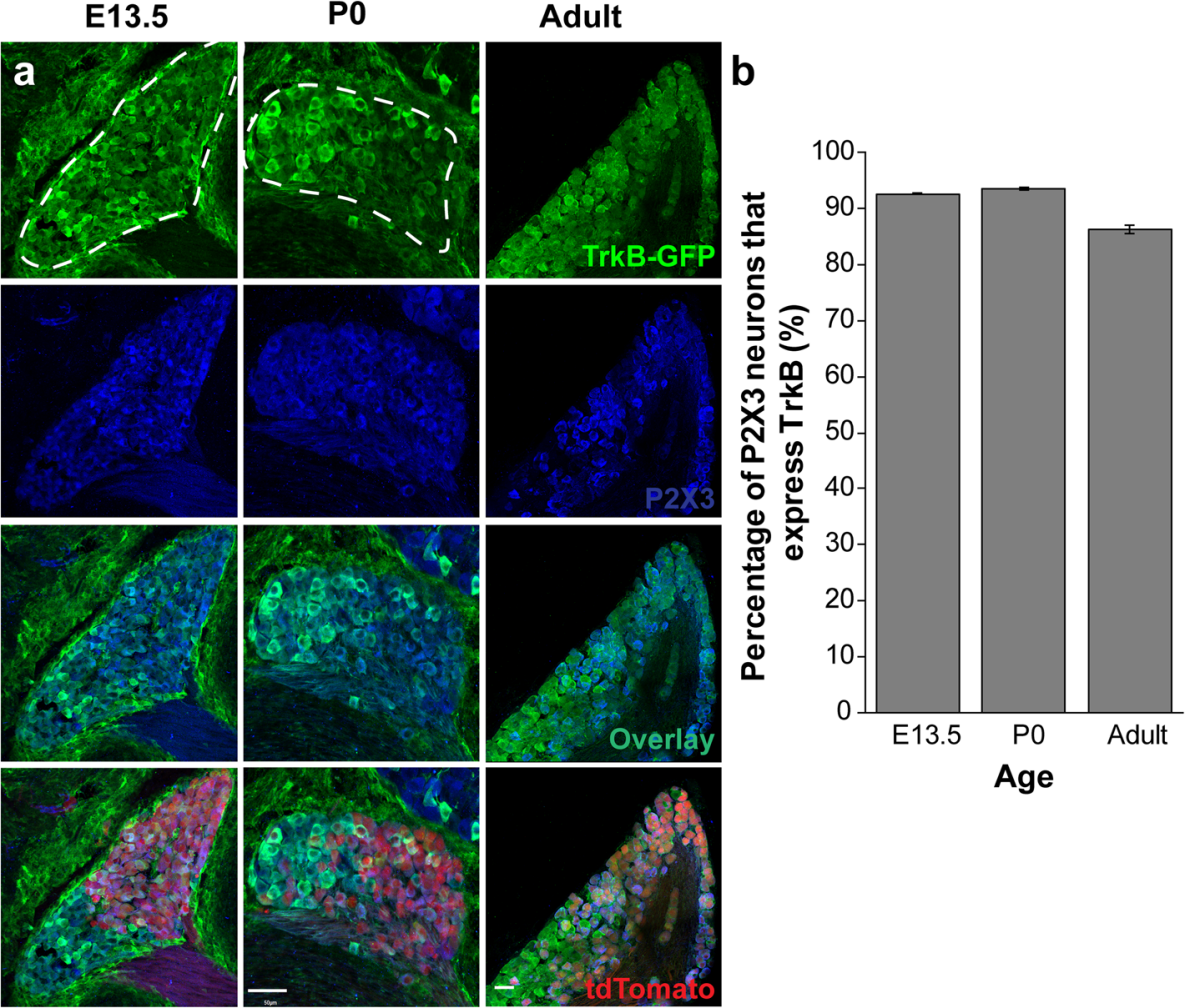
TrkB expression is consistent across ages in non-taste neurons. **a**) Representative image of geniculate ganglion sections labeled with TrkB^GFP^ (green), P2X3 (blue) and Phox2b-tdTomato (red) across ages (E13.5 (*n* = 2), P0 (*n* = 3), adult (*n* = 3)). Dashed lines illustrate the boundaries of the geniculate ganglion. **b**) Mean percentage ± SEM of non-taste neurons (Phox2b-, P2X3+) that express TrkB during development and adulthood. Scale bar = 50 μm

Because TrkB expression is reduced in tdTomato+ neurons, we next wanted to determine if this decrease was specific to oral sensory neurons, or if it also occurred in the auricular neurons of the geniculate ganglion. To determine if TrkB expression was also reduced in these auricular neurons during development, we examined TrkB expression in tdTomato- neurons. We used P2X3 (Fig. 3: blue) as a general marker of geniculate ganglion neurons [15, 33, 34]. Contrary to previous reports [33], P2X3 did not label all tdTomato- neurons (Fig. 3). P2X3 labeling was observed in many sensory neurons within the geniculate ganglion, including some Phox2b- cells. *TrkB*^GFP^ labeling was brighter in some regions of the geniculate ganglion, especially near Phox2b- neurons. This pattern continued into birth and adulthood. We counted the number of P2X3+/tdTomato- (non-taste neurons) with and without GFP (TrkB). Early in development (E13.5), most (92.9%) P2X3+ neurons also express *TrkB*^GFP^. Importantly, the percentage of P2X3+/tdTomato- neurons expressing TrkB did not significantly differ at birth (86.4%; *p* > 0.05) or adulthood (94.7%; p > 0.05). Thus, in somatosensory (non-taste) geniculate ganglion neurons, TrkB receptor expression remains consistent across development. We conclude that decreased TrkB expression during development is specific to a subset of neurons that carry oral sensory (primarily taste) information from the tongue and soft palate to the brain. TrkB reduction is likely part of the differentiation program for this specific subset of taste neurons.

### A small population of Phox2b + oral sensory neurons is TrkB-independent during development

When TrkB expression is eliminated during development, most geniculate ganglion neurons (94%) are lost by E13.5. However, 33% of the taste buds remain and most of these are innervated at birth [15], which seemed a surprising amount of innervation considering the large total geniculate neuron loss. Because neurons continue to migrate into the geniculate ganglion over a long period of embryonic development [20], we speculated that at least some of the decreased TrkB+ expression observed at E17.5 resulted from the migration of TrkB- neurons into the geniculate ganglion after E15.5. If this were the case, these TrkB+ tdTomato+ neurons would be present in the geniculate ganglion at later stages of development when TrkB is conditionally removed. Alternatively, TrkB expression may be downregulated after the critical developmental period for either geniculate neuron targeting or survival [8, 10, 21, 35]. In this case, most geniculate neurons would be lost in conditional TrkB knockouts. To evaluate these possibilities, we quantified the number of tdTomato+ neurons remaining in the geniculate ganglion at P20 in conditional TrkB knockout mice (Phox2b-Cre::tdTomato::*TrkB*^GFP/loxP^) compared to controls (Phox2b-Cre::tdTomato::*TrkB*^GFP/+^).

**Fig. 4:**
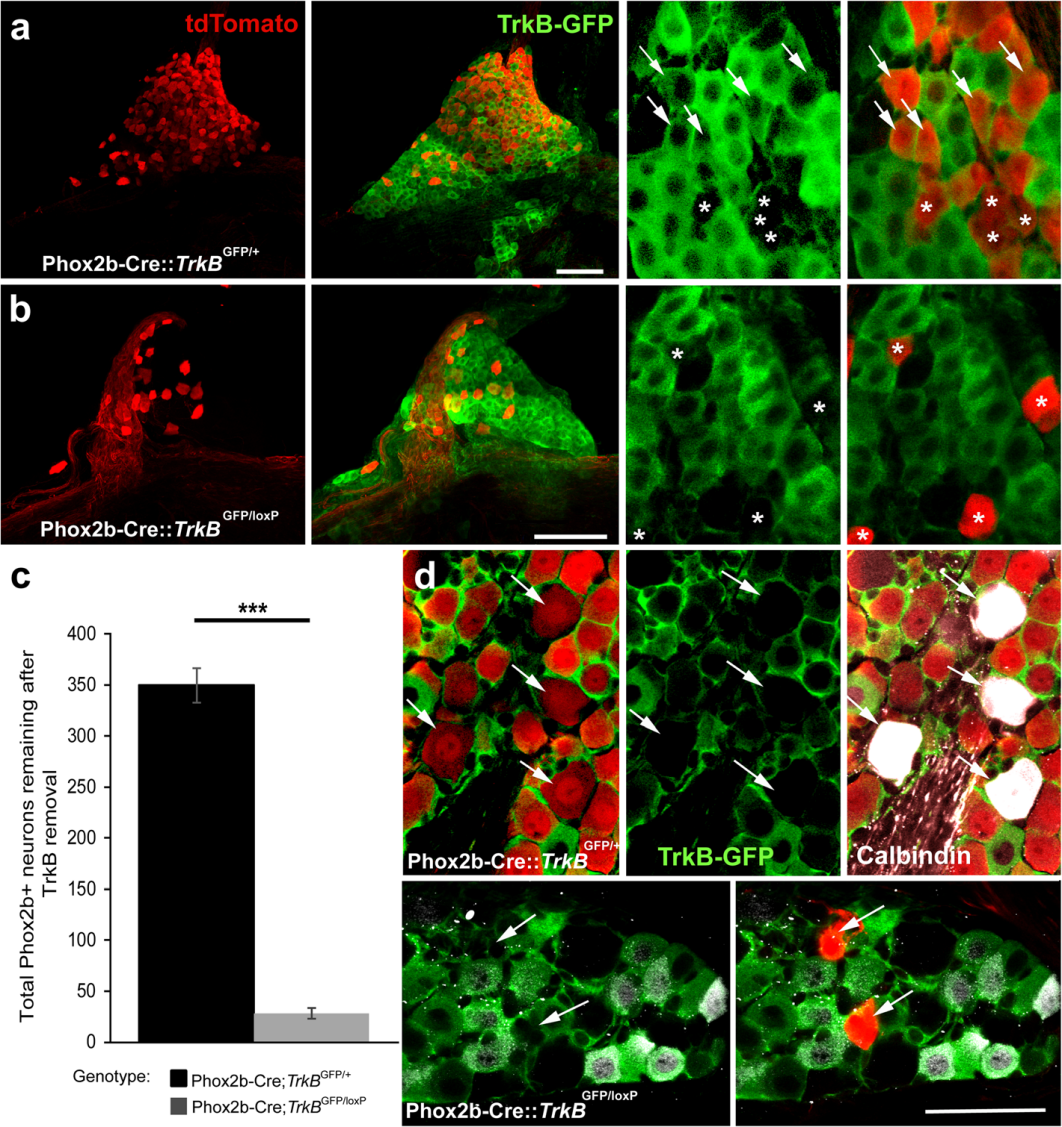
A small subpopulation of taste neurons (Phox2b-positive) do not depend on TrkB expression during development. **a**) A Z-stack composite image of a whole mount geniculate ganglion at P20 from Phox2b-Cre::tdTomato::*TrkB*^GFP/+^ (control). A higher magnification image of a single optical section from this ganglion illustrates that some tdTomato positive neurons also express GFP (arrows), while others do not (asterisks). **b**) Far fewer tdTomato-positive neurons can be observed in the whole mount of a geniculate ganglion at P20 from a Phox2b-Cre::tdTomato::*TrkB*^GFP/loxP^ (conditional knockout) mouse. A higher magnification image of a single optical section illustrates that tdTomato-positive neurons do not express GFP (asterisks). **c**) Mean ± SEM of Phox2b + neurons remaining after TrkB removal from oral cavity-projecting neurons (control, *n* = 5; conditional knockout, *n* = 8 conditional knockout). **d**) In control mice (Phox2b-Cre::tdTomato::*TrkB*^GFP/+^), a small number of brightly labeled calbindin positive neurons that also express tdTomato are present in the ganglion. Triple labeling with GFP (green), tdTomato (red) and calbindin (white) reveals that neurons double-labeled for both tdTomato and calbindin are not GFP positive (D, top arrows). Removal of TrkB from Phox2b positive neurons (Phox2b-Cre::tdTomato::*TrkB*^GFP/loxP^) results in a complete loss of this neuron population. Remaining tdTomato positive neurons do not express either calbindin or GFP (D, bottom, arrows). Scale bars in A and B = 100 μm, the scale bar in D = 50 μm and applies to all high magnification images. ***p < 0.001

As shown in adulthood (Fig. 1), TrkB expression was not homogenous across the geniculate ganglion at P20 (Fig. 4a, green). tdTomato expression was restricted to a subset of neurons, confirming that not all geniculate ganglion neurons express Phox2b (Fig. 4a, red). The geniculate ganglia from Phox2b-Cre::tdTomato:: *TrkB*^GFP/loxP^ mice were smaller and contained fewer tdTomato+ neurons relative to controls. Interestingly, an average of 25 ± 5.3 (*n* = 8) tdTomato+ neurons remained in the geniculate ganglion after TrkB removal compared to 349.8 ± 44.6 (*n* = 5) in control mice (Phox2b-Cre::tdTomato::*TrkB*^GFP/+^; Fig. 4c; ***p < 0.001). Of the 156 remaining Phox2b neurons in mice where GFP was quantifiable (*n* = 7) only one also appeared to be labeled with GFP (fewer than 1%). Therefore, the TrkB-independent population does not express either the full-length or the truncated TrkB receptor. This remaining 8% of tdTomato+ neurons represent a small subset of Phox2b-positive gustatory neurons. This proportion is equivalent to the percentage of tdTomato+/TrkB- neurons in the geniculate ganglion at E13.5 (5%). Therefore, new TrkB-independent taste neurons do not migrate into the geniculate ganglion after E13.5. These results support the conclusion that 92% of taste neurons express and depend on TrkB during development (E13.5 and earlier), and that TrkB is downregulated in a subset of these neurons between E15.5 and E17.5.

A recent study defined three subpopulations of gustatory neurons based on expression [24]. To test the relationship between one of these subpopulations and differential TrkB expression we labeled TrkB conditional knockout ganglion (Phox2b-Cre::tdTomato::*TrkB*^GFP/loxP^ *n* = 2) and control ganglia (Phox2b-Cre::tdTomato::*TrkB*^GFP/+^ *n* = 3) with calbindin, a marker for one of these subpopulations (Fig. 4d). Control ganglia (Phox2b-Cre::tdTomato::*TrkB*^GFP/+^) had 40 neurons double-labeled with calbindin and tdTomato (Phox2b), which represent 5% of the total population (Fig. 4e, top, arrows). None of these neurons expressed TrkB-GFP (Fig. 4e, top middle, arrows). There were no remaining neurons double-labeled with calbindin and tdTomato (Phox2b) in ganglia from conditional knockout mice (Phox2b-Cre::tdTomato::*TrkB*^*GFP/loxP*^, Fig. 4e, bottom, arrows). Consistent with a previous report [24], some of the Phox2b-negative neurons showed weak calbindin expression (Fig. 4d, bottom). Therefore, this small population of calbindin-positive oral cavity projecting neurons is a subset of the neurons that are TrkB-dependent during development, but lose their TrkB expression by adulthood.

### Remaining neurons innervate fungiform papillae but not taste buds, resulting in a substantial taste bud loss by P20

**Fig. 5:**
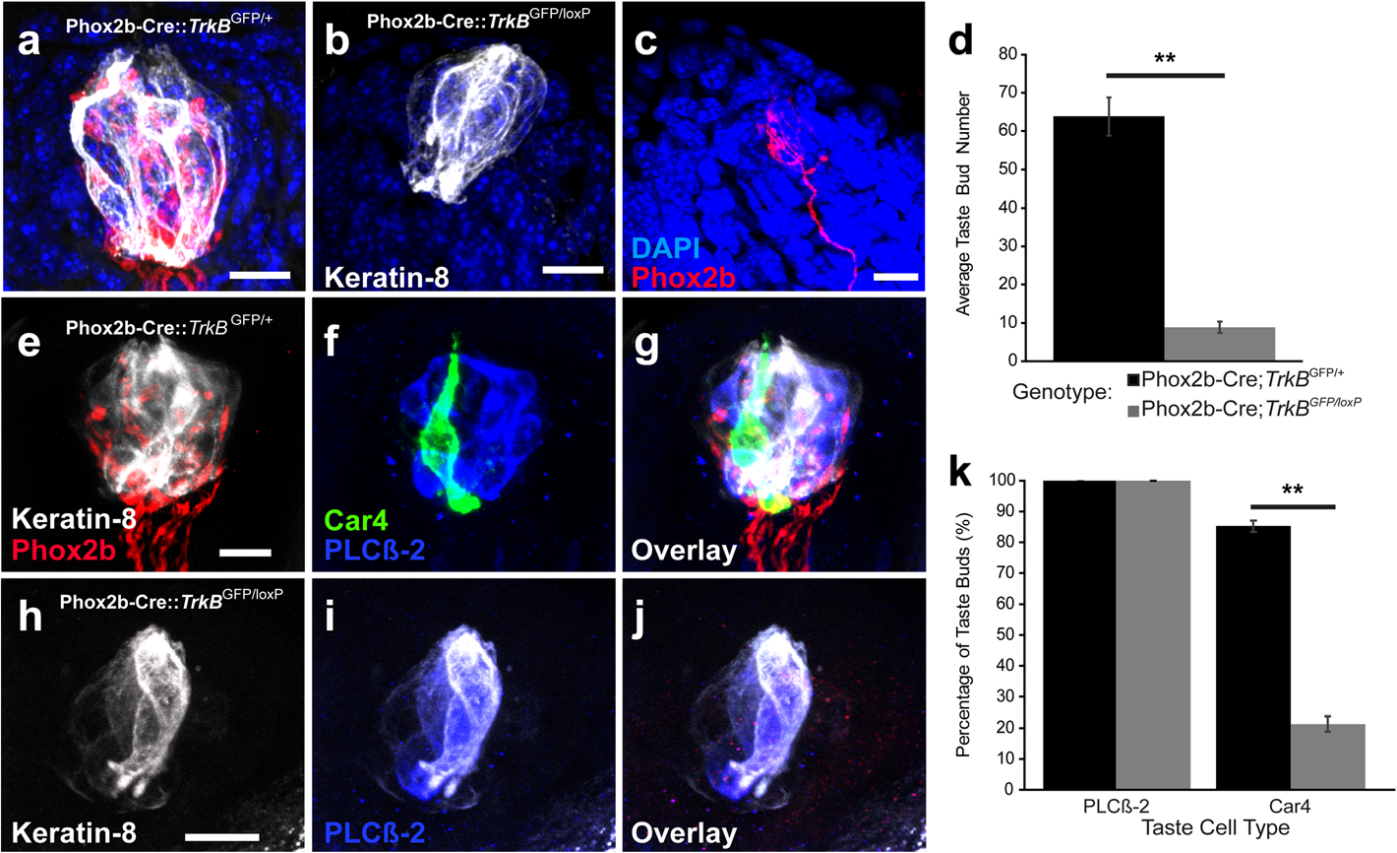
In conditional TrkB knockouts, remaining neurons innervate fungiform papillae, but not taste buds, resulting in substantial taste bud loss by P20. **a**) Representative image of a taste bud (keratin-8) with Phox2b + fibers (tdTomato) from Phox2b-Cre::tdTomato::*TrkB*^GFP/+^ mice. **b**) Representative image of a taste bud from Phox2b-Cre::tdTomato::*TrkB*^GFP/loxP^ lacking Phox2b-fibers. **c**) Phox2b + nerve fiber innervating a fungiform papillae (DAPI). D) Quantification of the number of taste buds remaining after TrkB removal from taste neurons (control, *n* = 3; conditional knockout, *n* = 5). **e**, **g**) Representative image of Phox2b-Cre::tdTomato::*TrkB*^GFP/+^ taste bud (keratin-8) with innervation (red) from Phox2b-Cre::tdTomato::*TrkB*^GFP/+^ mice, **f**, **g**) Car4+ (green) and PLCβ2+ taste receptor cells. H-J) Representative image of Phox2b-Cre::tdTomato::*TrkB*^GFP/loxP^ taste bud (keratin-8) with no innervation from Phox2b-Cre::tdTomato::*TrkB*^GFP/+^ mice and I) PLCβ2+ taste receptor cells K) Quantification of percentage of taste buds with PLCβ2+ cells and Car4+ cells. Data represent the mean of percentage ± SEM. Scale bar = 10 μm. Scale bar on E applies to F and G. Scale bar on H applies to I and J. **p < 0.01

A small group of Phox2b + geniculate neurons do not depend on the TrkB receptor during development. These oral sensory neurons are primarily gustatory, but also include a small population of somatosensory neurons [24, 36]. Therefore, whether the remaining neurons in conditional TrkB knockouts innervated taste buds was unclear. To examine the location of innervation for these remaining oral sensory neurons, we examined the entire tongue for dsRed positive nerve fibers in control (Phox2b-Cre::tdTomato::*TrkB*^GFP/+^; Fig. 5a) and conditional TrkB knockout (Phox2b-Cre::tdTomato::*TrkB*^GFP/loxP^; Fig. 5b, c) mice. Conditional TrkB removal reduced taste bud size and completely eliminated tdTomato innervation within taste buds (Fig. 5b). We found only a few tdTomato+ fibers innervating the lingual epithelium. These fibers innervated fungiform papillae, but these papillae lacked taste buds (Fig. 5c). The few neurons remaining following TrkB removal might belong to a somatosensory neuron population, since they innervated fungiform epithelium and not taste buds.

TrkB is required for the survival of 92% of oral sensory neurons in the geniculate ganglion; gustatory neurons are required to maintain taste buds [37–40]. So, next we quantified the number of taste buds that developed in the absence of TrkB fibers. Consistent with previous findings, TrkB removal significantly reduced the number of taste buds (Phox2b-Cre::tdTomato::*TrkB*^GFP/+^: 63.8 ± 4.9 vs. Phox2b-Cre::tdTomato::*TrkB*^GFP/loxP^: 8.9 ± 1.5, **p < 0.01, Fig. 5d). TrkB removal from Phox2b + neurons reduced taste buds by 86%, suggesting that TrkB-dependent neurons support most taste buds during development.

Because the remaining taste buds completely lacked innervation, we also wanted to determine if nerve fibers preferentially supported specific subtypes of taste receptor cells. Taste buds from Phox2b-Cre::tdTomato::*TrkB*^GFP/+^ (Fig. 5a, e-g) and Phox2b-Cre::tdTomato::*TrkB*^GFP/loxP^ (Fig. 5b, h-j) mice were labeled for PLCβ2, a marker for Type II taste receptor cells that transduce bitter, sweet, umami [41], and for Car4, a marker of Type III cells that transduce sour [42]. We quantified the percentages of taste buds with PLCβ2+ and Car4+ cells for both genotypes. All taste buds from Phox2b-Cre::tdTomato::*TrkB*^GFP/+^ mice contained both Car4+ and PLCβ2+ taste receptor cells (Fig. 5f and g). In contrast, taste buds from conditional knockouts typically lacked Car4+ cells (Fig. 5i and j), while PLCβ2+ cells were present in 100% of taste buds examined from both genotypes (Fig. 5k). Conditional TrkB knockout mice had significantly fewer Car4+ cells in the remaining taste buds to ~ 21.3 ± 2.5% compared to controls (Phox2b-Cre::tdTomato::*TrkB*^GFP/+^: 85.3 ± 1.8%, **p < 0.01, Fig. 5k). These data suggest that the loss of innervation caused by TrkB removal markedly reduces the number of taste buds and Type III taste receptor cells by P20.

## Discussion

During development, the neurotrophin BDNF and its receptor TrkB play vital roles in the development of geniculate ganglion neurons [8, 43]. Specifically, BDNF and NT4 regulate apoptotic developmental cell death and targeting via TrkB [3, 8, 15, 44, 45]. Most geniculate neurons express TrkB in early development (E13.5 [15, 17]). By adulthood, however, the ligand for TrkB, BDNF, is expressed in a subset of taste receptor cells, and BDNF removal reduces some but not all of the nerve fibers innervating taste buds [21, 22]. These findings suggest that by adulthood, TrkB expression may be limited to a subset of taste neurons and BDNF-TrkB signaling functions to maintain innervation of this subset, but not others [21, 22]. Consistent with these findings, we found that not all adult Phox2b + (taste) neurons expressed TrkB. Furthermore, TrkB expression decreased in these neurons between E15.5 and E17.5 such that it was expressed in half the taste neurons by adulthood. This reduction likely signifies a changing role for TrkB during development.

There were two potential explanations for how this developmental reduction in TrkB expression occurred. One possibility was that TrkB receptors were downregulated and became restricted to a subset of geniculate neurons. Alternatively, TrkB expression could have decreased because TrkB- neurons migrate into the geniculate ganglion after E13.5 [20], an age when most geniculate neurons both express and depend on TrkB [15]. We found that TrkB- neurons do not migrate into the geniculate ganglion at later embryonic ages, and instead the TrkB receptor is downregulated. Specifically, TrkB expression did not decrease until after E15.5, which is later than might be expected if new TrkB-expressing neurons continue to migrate into the geniculate ganglion. Furthermore, the small number of TrkB-expressing oral cavity neurons present in the E13.5 geniculate ganglion (approximate 5%) accounted for most of the remaining neurons in the conditional TrkB knockout at P20 (approximately 9%). We conclude that most (91%) of the oral cavity neurons initially express and depend on the TrkB receptor and then 41% of these neurons downregulate TrkB between E15.5 and E17.5.

The timing of TrkB downregulation in the oral cavity-projecting neurons corresponds with the timing of decreased BDNF expression in the taste bud [32]. Around E14.5, geniculate neurons depend on BDNF expression in the lingual epithelium to properly innervate their targets [8, 11]. By E15.5, geniculate neurons have already reached their targets, so BDNF should no longer be needed in the tongue at such high levels. Also, at these later stages, geniculate neurons no longer depend on BDNF for survival [10]. Thus, the timing of decreased TrkB expression corresponds with changing roles of BDNF-TrkB signaling during development. Another example in these same neurons of a developmentally critical gene changing roles during development, is the growth factor receptor, Ret (27). Ret regulates expression of the transcription factor, Phox2b, embryonically, but later identifies a subset of adult Ret expressing neurons that are likely somatosensory in function [27]. The role of TrkB in adulthood may be to regulate plasticity and branching characteristics for a subset of oral sensory geniculate neurons (TrkB-expressing [22]), but could also regulate taste function in this same neuron subset [14].

In the dorsal root ganglion, a series of developmentally expressed transcription factors and growth factor receptors orchestrate neuron subtype development [46]. However, our current understanding of neuron subtypes and how they differentiate within the geniculate ganglion is still emergent. The transcription factor Phox2b plays a role in the differentiation of neurons that control viscero-sensory functions [47–49] and so likely specifies oral sensory from auricular neurons in the geniculate ganglion [23, 24]. We found that the TrkB expression is downregulated specifically in this subset of geniculate neurons (Phox2b + oral sensory neurons), but not in the auricular neurons. A finding consistent with a higher level of TrkB expression in auricular neurons than oral sensory neurons by adulthood [24]. Therefore, Phox2b-regulated differentiation into an oral sensory neuron subtype is likely required for this decrease in TrkB expression. However, this decrease only occurs in half of the Phox2b + neurons. Given that Phox2b expression first occurs at E9–9.5 [49, 50], which is days before the downregulation of TrkB, Phox2b likely initiates a cascade of events orchestrating the differentiation of taste neurons followed by differentiation of neuron subtypes. A complex series of events such that combinations of these factors work together to orchestrate adult expression patterns (24) seems likely. For example, the growth factor receptor, Ret, is expressed in both TrkB+ and TrkB- neurons, which further divides oral sensory neurons into Ret+TrkB+,Ret+TrkB-,Ret-TrkB+, etc. [27]. Using a combination of factors to specify neuron subtypes permits a smaller number of factors to orchestrate the development of a larger number of neuron subtypes (i.e. two factors can specify four types, etc). It is now clear that multiple molecular subtypes of oral sensory neurons are present in the geniculate ganglion [24], although precisely how many is still unclear.

Interestingly, a recently defined subpopulation of taste neurons that express calbindin [24], are a subset of the neurons that depend on TrkB during development but lose TrkB-expression by adulthood. This finding is consistent with the idea that adult TrkB+ and TrkB- neurons are too large to be a single pure neuronal subset. However, it is also consistent with the idea that pure neuronal subsets do not contain both TrkB+ and TrkB- neurons in adulthood. Instead, the calbindin+ subpopulation is one of multiple gustatory neuron subpopulations whose adult innervation patterns are not likely to be regulated by BDNF [21, 22].

While much of gustatory neuron differentiation may be regulated by a cascade of intrinsically expressed molecular factors, the target also likely regulates gustatory neuron differentiation. Once taste neurons innervate their targets BDNF from the taste bud could maintain TrkB expression in gustatory neurons, such that those innervating BDNF-expressing taste receptor cells retain the higher TrkB levels. Because BDNF is preferentially expressed in the taste receptor cells that express the neurotransmitter serotonin [14], TrkB might be preferentially expressed in neurons that have the serotonin receptor, Htr3a, which mediates the neuronal serotonin response [51]. Consistently, mean TrkB expression is higher in the Phox2b + neurons that express Htr3a relative to those that do not [24].

When TrkB was conditionally removed from Phox2b + neurons during early development, a few remaining neurons innervated the oral cavity. This is consistent with studies using full TrkB knockouts reporting that TrkB removal substantially reduces the number of geniculate ganglion neurons [15, 18]. However, these previous studies could not examine innervation patterns of these remaining neurons, as they lacked appropriate genetic markers. Here, we found a few tdTomato+ fibers innervating the epithelium in a fungiform papilla and no labeled innervation in remaining taste buds. There are several possible sources of these remaining fibers. These few remaining nerve fibers might have originally innervated locations previously occupied by a taste bud and remain when taste buds are lost. Alternatively, these remaining neurons may be non-taste, and could belong instead to a small somatosensory population in the geniculate ganglion that innervate the oral cavity [27, 36, 52]. We found this neuron population to be surprisingly small (only 4 neurons in some individuals); therefore, it may not be a large enough population to have been identified in recent expression studies [24].

Consistent with the neuronal loss, conditional TrkB knockout mouse tongues had fewer taste buds. Previous reports of full TrkB knockouts indicate that 37% of the taste buds remained [15], compared to only 14% in the current study. Furthermore, in one of these earlier studies, 58% of the remaining taste buds were innervated in full TrkB knockout mice [15], while we observed no remaining taste bud innervation. One explanation for these discrepancies is that P2X3 was previously used as marker for taste bud innervation [15]. P2X3 is expressed in some somatosensory neurons [53]; therefore, non-taste P2X3+ fibers may innervate some of the remaining taste buds. More likely, these two studies examined different postnatal ages; therefore, the remaining Phox2b + neurons in the geniculate ganglion might initially innervate taste buds at birth but retract by P20, resulting in an additional postnatal taste bud loss.

Taste buds require innervation to retain their normal size and integrity [40, 54, 55]. Because some taste buds remained despite complete innervation loss by P20, we investigated whether developmental denervation influences some taste cell types more than others. We found that Car4+ taste receptor cells, known to express SNAP25 and have synapses [56, 57], were absent from most remaining taste buds. Alternatively, all remaining taste buds still contained PLCβ2+ taste receptor cells. Therefore, Car4+ taste receptor cells may depend on innervation more than PLCβ2+ receptor cells. Innervation likely supports taste buds at least in part by releasing sonic hedgehog [58, 59]. However, in the absence of innervation, the few remaining taste buds could be supported by epithelial-derived sonic hedgehog [28, 58, 60–62].

## Conclusions

Taken together, these data show that TrkB expression and dependence divides taste neurons into subpopulations. We suggest that BDNF expression initially guides TrkB+ fibers to innervate taste buds during a critical developmental period [8]. During this time, all taste neurons express and depend on TrkB, while the 9% of oral cavity-projecting neurons that are TrkB-independent are oral somatosensory. After this critical period, TrkB expression in Phox2b + neurons is downregulated, dividing these neurons into a TrkB+ and TrkB- subpopulations. In the adult taste system, BDNF likely maintains TrkB expression and taste bud innervation of the TrkB+ but not the TrkB- taste fibers [22]. These TrkB+ taste neurons may play a different functional role than TrkB- neurons in adulthood. Because taste neuron subclasses are likely defined by combinations of differential gene expression, TrkB likely joins with other factors [24, 27, 63, 64] to separate taste neurons into types and influence their ability to innervate subclasses of taste receptor cells.

## Abbreviations

BDNF: brain derived neurotrophic factor
DAPI: 4,6-diamidino-2-phenylindole dihydrochloride
K8: cytokeratin-8
PB: phosphate buffer
PBS: phosphate-buffered saline.
PBST: PBS containing 0.5% Triton X-100
PFA: paraformaldehyde
SEM: standard error of the mean
TrkB: tropomyosin kinase B receptor
